# Methodologies for the Extraction of Phenolic Compounds from Environmental Samples: New Approaches

**DOI:** 10.3390/molecules14010298

**Published:** 2009-01-09

**Authors:** Cristina Mahugo Santana, Zoraida Sosa Ferrera, M. Esther Torres Padrón, José Juan Santana Rodríguez

**Affiliations:** Department of Chemistry, Faculty of Marine Sciences, University of Las Palmas de Gran Canaria, 35017, Las Palmas de Gran Canaria, Spain; E-mails: cristina.mahugo101@doctorandos.ulpgc.es (C-M. S.), zsosa@dqui.ulpgc.es (Z-S. F.), mtorres@dqui.ulpgc.es (M-T. P.)

**Keywords:** Phenolic compounds, Solid-phase extraction, Solid-phase microextraction, Microwave assisted extraction, Micellar medium

## Abstract

Phenolic derivatives are among the most important contaminants present in the environment. These compounds are used in several industrial processes to manufacture chemicals such as pesticides, explosives, drugs and dyes. They also are used in the bleaching process of paper manufacturing. Apart from these sources, phenolic compounds have substantial applications in agriculture as herbicides, insecticides and fungicides. However, phenolic compounds are not only generated by human activity, but they are also formed naturally, e.g., during the decomposition of leaves or wood. As a result of these applications, they are found in soils and sediments and this often leads to wastewater and ground water contamination. Owing to their high toxicity and persistence in the environment, both, the US Environmental Protection Agency (EPA) and the European Union have included some of them in their lists of priority pollutants. Current standard methods of phenolic compounds analysis in water samples are based on liquid–liquid extraction (LLE) while Soxhlet extraction is the most used technique for isolating phenols from solid matrices. However, these techniques require extensive cleanup procedures that are time-intensive and involve expensive and hazardous organic solvents, which are undesirable for health and disposal reasons. In the last years, the use of news methodologies such as solid-phase extraction (SPE) and solid-phase microextraction (SPME) have increased for the extraction of phenolic compounds from liquid samples. In the case of solid samples, microwave assisted extraction (MAE) is demonstrated to be an efficient technique for the extraction of these compounds. In this work we review the developed methods in the extraction and determination of phenolic derivatives in different types of environmental matrices such as water, sediments and soils. Moreover, we present the new approach in the use of micellar media coupled with SPME process for the extraction of phenolic compounds. The advantages of micellar media over conventional extractants are reduction of organic solvent, low cost, easy handling and shorter time procedures.

## Introduction

Phenolic compounds are present in the environment as a result of their uses and the processes in which they are implicated. Although they can be originated naturally due to the degradation of humic substances, tannins and lignins, many industrial processes, including production of drugs, textiles, dyes, pesticides and paper, are the main source of these compounds in the environment [1,2]. Furthermore, chlorophenols have been widely used as wood preservative agents and disinfectants during decades, so they have been released in the environmental media [3]. Although the use of pentachlorophenol is prohibited in most countries, it is still widely found in the wood of pallets, containers, crates and in cardboard, paper, etc. Wooden crates and cardboard boxes are often used to store and transport fresh fruits. Consequently, chlorophenols present in these materials may contaminate the stored fruits by migration [4]. Chlorinated phenols can also be generated from non-chlorinated phenols during drinking water chlorination [5]. Tri-, tetra- and pentachlorophenol are considered the precursors in the formation of corresponding chloroanisoles, known to be powerful odorants in corks and wine. This is one of the most critical problems in the enological industry [6]. Nitrophenols are formed photochemically in the atmosphere from vehicle exhausts [7]. More hydrophilic phenols, such as less chlorinated phenols, are easily distributed in the aquatic media, meanwhile non polar compounds, more chlorinated phenols such as pentachlorophenol, usually persist longer in environment, especially in soils and sediments.

**Figure 1:**
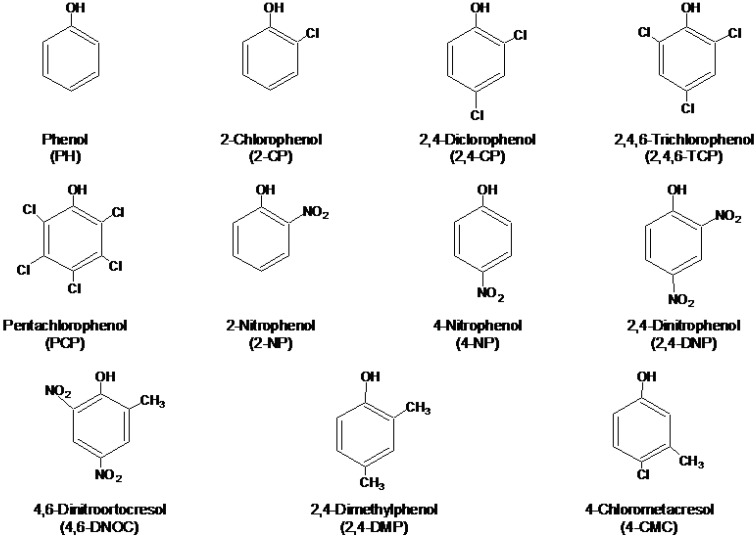
Structures of eleven phenolic compounds considered priority pollutants by US EPA.

Phenols, and particularly chlorophenols, are toxic, and potentially carcinogenic, and they can affect the taste and odour of drinking water with concentrations as low as a few µg·L^-1^. As a consequence, both, the US Environmental Protection Agency (EPA) and the European Union (EU) have included some phenols, mainly chlorophenols and nitrophenols, in their lists of priority pollutants [8,9]. The structures of eleven phenols considered priority pollutants by the EPA are shown in Figure 1. EU Directive 2455/2001/EC sets a maximum concentration of 0.5 µg·L^-1^ in drinking water and their individual concentration should not exceed 0.1 µg·L^-1^.

Analytical techniques commonly used in the determination of phenols are high-performance liquid chromatography (HPLC) and capillary electrophoresis (CE) in combination with ultraviolet detection (UV), electrochemical detection or mass spectrometry detection (MS) [10,11,12,13,14]. Liquid chromatography of phenols is generally carried out with the addition of acids or buffers to the mobile phase. Their function is to suppress the ionisation of both, the analytes and the residual silanols of the stationary phase base material, which otherwise would either decrease retention on the analytical column or lead to interactions of the analytes and the stationary phase, resulting in lower separation efficiencies.

Also, gas chromatography (GC), using several detection methods like flame ionisation detection (FID), electron-capture detection (ECD) or mass spectroscopy detection (MS), have been used, although in the case of CG, a derivatization step is needed [15,16,17]. The high polarity of free phenols hinders their correct chromatographic resolution because they produce broad, tailed peaks; this limitation can be circumvented by derivatising free phenols to less polar compounds, such as acetylated derivatives. They are commonly derivatised either before or after extraction, or with an on column reagent in the GC injector port.

Nevertheless, the detection limits imposed by environmental quality legislation can only be achieved by using appropriate sample preparation techniques, which provide high enrichment factors of these analytes. Current official analytical methods for phenolic compounds extraction are liquid-liquid extraction (LLE) (US EPA Methods 604, 605, 8041) [18,19,20] for liquid samples, and Soxhlet extraction, for solid samples [21]. These methods require expensive and hazardous organic solvents, which are undesirable for health and disposal reasons, and they involve a long time per analysis. For these reasons, these traditional extraction sample methods have been replaced for other methodologies, more sensitive, selective, fast and environmentally friendly.

This overview reports a description of the different techniques used to date to extract and preconcentrate phenolic derivatives from liquid and solid environmental samples. We present the main advantages and limitations of more conventional methods, those which use organic solvents, and highlight recent developments and trends in this field with new methodologies, namely green methods or environmentally friendly methods, which try to eliminate or reduce, at least, the use of these solvents.

## Extraction methods using organic solvents

### Liquid samples preparation

As mentioned above, the current official analytical methods used to extract phenolic compounds from liquid samples are based on liquid-liquid extraction (LLE), followed by gas chromatography (GC) determination with different detection methods. Although this technique offers efficient and precise results, it is relatively time-consuming, possibly harmful due the use of large volume of organic solvents (frequently toxic) and highly expensive. For these reasons, there is an increasing tendency to replace LLE by solid-phase extraction (SPE) for liquid samples. SPE was developed in the 1980s, and has emerged as a powerful tool for chemical isolation and purification. This methodology is an alternative extraction to LLE due to it reduces organic solvents consumption, the length of analysis and it can be automated [22,23,24].

This extraction method is based on differential migration processes, during which analytes are adsorbed in a solid sorbent and then eluted by elution solvent. Cartridges, columns and syringes are classic devices used in SPE. Elution of retained analytes into the sorbents is carried out with different organic solvents like ethyl acetate, methanol, acetonitrile or acetone [25,26,27]. The type of solvent chosen depends on the kind of sorbent and the polarity of each analyte. SPE technique has been developed in the off-line and on-line modes, although on-line approach is preferred due to advantages such as higher sensitivity and less manipulation of the samples.

Non-polar reversed-phase sorbents with silica base were the first supplies used in SPE of phenolic compounds from water samples [28,29,30]. Among these types of sorbents, C_18_ was the most accepted. However, these types of sorbent were soon replaced by other ones due to the low efficiencies achieved in the extraction of phenols. First of all, the interactions between phenolic compounds and silica sorbent are apolar Van der Waals interactions, therefore, the pH sample must be adjusted to avoid ionisation of some phenols, such as for pentachlorophenol and dinitrophenols, but silica based materials exhibit pH instability. On the other hand, phenol and monosubstituted phenols are hydrophilic compounds, with lower affinity for non-polar sorbents, such as silica sorbents, and they present low breakthrough volume. This is the volume of analytes solution that can flow through the sorbent before breakthrough occurs and it is a measure of the sorbent extraction capacity. In SPE, the breakthrough volume is an important feature to take into account since it determines the detection limit that can be reached for each compound. Galcerán et al. obtained recoveries about 70%, except for phenol and o-chlorophenol, for 10 µg·L^-1^ samples when used C_18_ cartridges. The breakthrough volumes for these two compounds were 20 and 100 mL, respectively, while all other compounds of lower polarity had breakthrough volumes of over 250 mL [31].

For these reasons, there was a trend to use more stable organic polymer sorbents. In this way, new sorbents have been developed by modification of polystyrene-divinylbenzene polymeric (XAD) resins and they have been incorporated to SPE devices. They comprise a polystyrene-divinylbenzene (PS-DVB) hydrophobic structure with different particle size, superficial areas and crosslinked grades. The procedure to extract phenols from water samples with this type of sorbents is similar to the procedure used with silica sorbents. The large carbon content of these sorbents (90% for polymeric sorbents whereas 18% for C_18_), the higher surface area, compared to silica-based materials, and the presence of aromatic modifiers in their surface result result in greater breakthrough volumes, up to 1 L of water for some sorbents [32,33,34].

Among these types of cartridges, OASIS (*N*-vinylpyrrolidane-divinylbenzene based sorbent) is the most used. De Almeida *et al*. developed an off-line solid-phase extraction procedure to extract priority pesticides and priority organic pollutants, including some phenolic compounds, from river waters using this type of cartridge. Recoveries for the majority of compounds were between 70 and 130% with standard deviations below 30%. All the compounds were studied in a concentration range from 0.05 to 2 mg·L^-1^ for 200 mL of river water [35]. The same type of cartridges was used by Wissiack *et al*. to extract the U.S. EPA eleven priority pollutants phenols from spiked river water samples [36]. The recoveries obtained with the on-line SPE HPLC-APCI-MS technique were ranged between 90 and 105%. Limits of detection ranged from 40 to 280 ng·L^-1^ and relative standard deviation below 8% were obtained for 10 mL of water samples for concentrations about one order of magnitude above of LODs.

Another polymeric cartridge, LiChrolut EN, has been used successfully to extract phenolic compounds from aqueous samples [37]. Cheung *et al*. used them for the extraction of eight phenolic compounds in effluent from various tertiary sewerage treatment plants with good recoveries for analytes investigated which differed widely in polarity. Recoveries were found to remain high, even for samples consisting of large amounts of organic matter. The SPE procedure described in this work was able to provide detection limits at ppt levels for phenol and the chlorophenols tested [38].

Graphitised carbons are the third kind of sorbent used in SPE of phenolic compounds [39]. In this case, it is not necessary to acidify water samples to extract phenols, because of the strong interactions of phenolates with the positively charged sites on the carbon surface. Di Corcia *et al*. developed a procedure for determining the U.S. EPA eleven priority pollutant phenols in natural waters [40]. The method involves passing 1 and 4 L, respectively, of drinking and river waters through a reversible cartridge filled with 0.5 g of graphitized carbon black (Carbograph 4) at flow-rates of about 100 mL·min^-1^. Phenols were separated and quantified by ion-suppression, reversed-phase LC with UV detection. Recoveries of phenols, including phenol itself, were higher than 90%.

Although SPE is widely accepted, as an alternative extraction and clean-up method to LLE, has certain limitations, especially for analytes such as phenolic derivatives, which exhibit different behaviour in terms of polarity and acidity. Solid phase micro-extraction (SPME) has been developed to resolve some of the drawbacks that present the SPE technique. This extraction method, introduced by Pawliszyn in 1990 [41], accomplishes the sampling, extraction and enrichment in a single step process. This approach consists of a fused-silica fiber coated with different polymers, which is housed in the needle of a syringe. SPME is based on equilibrium between the analyte concentration on the sample and that in the fiber coating.

For SPME, two possible approaches have been evaluated: headspace exposure and direct immersion of the fiber on the aqueous sample [42,43,44]. However, although SPME of phenols from the water headspace minimises fiber contamination by adsorption of non volatile matrix components, this approach is limited to volatile compounds, typically up to tri-chlorinated phenols.

Desorption of analytes can be carried out by placing the fiber in the hot injector of the gas chromatograph, where the analytes are thermally desorbed. In liquid chromatography, a SPME-HPLC interface or desorption chamber can be used to desorb the analytes. Two modes of desorption are possible: dynamic desorption and static desorption. In the dynamic mode, analytes are desorbed from the fiber by the moving stream of the mobile phase. In the static mode, the fiber is kept in the desorption chamber, filled with an organic solvent or the mobile phase, for a period of time. These both procedures are called on-line desorption mode. But analytes desorption from the fiber may be also made by off-line desorption using a glass vial with a conical glass inside filled with an organic solvent. Peñalver *et al*. applied SPME-HPLC to compare dynamic and static modes of desorption in the extraction of eleven phenolic compounds considered priority pollutants by the US Environmental Protection Agency from the aqueous samples. Results obtained demonstrated that static desorption achieved better recoveries for these compounds [45].

The first fibers used in extraction of phenols were polydimethylsiloxane (PDMS) and polyacrylate (PA). Results obtained with the PDMS fiber were not satisfactory due to the relative non-polar nature of this coating, whereas the more polar PA fiber was found more suitable for the extraction of phenolic compound as it has a great affinity for these compounds, mainly for the more polar ones [46,47,48]. However, when using PA fiber in SPME-HPLC, phenolic compounds are not totally desorb from the fiber during the desorption step, with the exception of pentachlorophenol. Other polar fibers, such as polydimethylsiloxane-divinylbenzene (PDMS-DVB) or carbowax-templated resin (CW-TPR), have been tested to extract phenolic compound from water samples. In this case, the most polar fiber coating, CW-TPR, led to higher extraction recoveries than PDMS-DVB [49,50,51,52].

The main stage in the SPME optimisation is the fiber choice, but after that, there are some parameters that have to be controlled in this technique to guarantee good extraction efficiencies. Among these variables are time and temperature of adsorption, pH and ionic strength of the sample and parameters affecting the desorption process, such as time and composition of the desorption solvent [50,51,52]. Compounds with low diffusion coefficients have long equilibration times; in this case to abbreviate the analysis time, an extraction–time curve is constructed, showing the dependence of the amount of the analyte extracted as a function of time. The shortest acceptable time is chosen according to time must be very well controlled to ensure reproducibility. González-Toledo *et al*. applied SPME-HPLC to the analysis of priority pollutant phenolic compounds in water samples and they found that the most polar phenols, such as phenol or 2-chlorophenol, reached equilibrium in 10–20 min, whereas the less polar ones, like 2,4,6-trichlorophenol or pentachlorophenol, require much longer equilibration times, up to 80 min [52].

Agitation is normally used to achieve faster equilibration because it enhances the diffusion of analytes towards the fiber. The effectiveness of the agitation technique determines the equilibration time of aqueous samples. There are different agitation methods in SPME, namely, magnetic stirring, vortex technique and sonication. Magnetic stirring, which requires a stirring bar in the vial, is most commonly used in SPME due to its availability in analytical laboratories. Moreover, it can be used in different SPME sampling modes.

The extraction temperature has two opposing effects on the SPME technique. Increasing temperature enhances the diffusion coefficient of analytes; on the other hand, as the adsorption is an exothermic process, increasing temperature reduces the distribution constant of the analytes. In the case of phenols, highest relative responses for mono and dichlorophenols have been obtained between 30 and 40 ºC, whereas the affinity of the analytes for the fiber coating increases with temperature for the most chlorinated phenols, being maximum in the range 50–60 ºC [50]. In general, a compromise value of 40-50 ºC is frequently chosen to achieve the extraction of phenols in SPME [45,51,53].

Electrolytes are usually added to the samples in SPME experiments to improve extraction of organic compounds from aqueous solutions. The addition of salts, such as sodium chloride or sodium sulphate, increases the ionic strength of the solution and this makes organic compounds less soluble, increasing the partition coefficients several times [45,53]. The effect of ionic strength on the recoveries of phenols was studied by Sarrión *et al*. [51]. They found that the extraction of chlorophenols in water samples was enhanced by the addition of NaCl, obtaining highest increase mainly for more polar compounds. The same result was reported previously by Buchholz and Pawliszyn [54].

The pH of the sample is important for slightly acid or basic compounds, e.g., phenols, because they need to be kept in the undissociated form. The effect of pH on the sorption of phenols depends on their pK_a_. For example, compounds with high pK_a_ values, such as mono, and most of the dichlorophenols, showed no significant change in the amount absorbed when the pH was varied from 7 to 2.5. However, for compounds with pK_a_ values between 4.7 and 7, the decrease in pH produced an increase in the responses [45,51,53]. Therefore, pH must be adjusted between 2-4 to ensure that all phenols are in their neutral form, which increases affinity for the fiber coating. Anyway, it is necessary take account the type of fiber when the pH solution is modified to prevent its degradation.

Respect to the solvents utilised in the desorption step, it depends on the mobile phase employed in the SPME-HPLC procedure, but methanol, acetonitrile or mixture of both solvents are the most used to desorb the phenolic compounds from the fiber [45,51,52,53,54].

On the other hand, miniaturised LLE, or liquid-phase microextraction (LPME), was introduced in 1996 to facilitate automation and to reduce the consumption of organic solvents. This technique involved the use of a droplet of organic solvent hanging at the end of a micro-syringe needle. This organic micro-droplet was placed in an aqueous sample, and the analytes present in the aqueous sample were extracted into the organic microdroplet [55]. Subsequently, the organic micro-droplet was withdrawn into the syringe and the microextract was injected to a capillary gas chromatograph (CGC), for the analysis. However, LPME based on hanging droplets is not very robust, and the droplets may be lost from the needle tip of the syringe during extraction. Recently, an alternative concept for LPME, based on the use of porous hollow fibers, has been introduced [56,57]. In this new approach, analytes of interest are extracted from aqueous samples through a thin layer of organic solvent, immobilized within the pores of a porous hollow fiber, and into an acceptor solution inside the lumen of the hollow fiber. Afterwards, the acceptor solution is directly subjected to a final analysis by CGC, high-performance liquid chromatography (HPLC), capillary electrophoresis (CE), or mass spectrometry (MS) [58]. Li-Wen Chung *et al*. [59] have determined chlorophenols in environmental samples using liquid-phase microextraction (LPME) coupled with gas chromatography–mass spectrometry (GC–MS). A polypropylene hollow fiber filled with 1-octanol was immersed in 30 mL of donor solution (15 mL of sample solution and 15 mL of pH buffer solution) during 80 minutes. Under the optimal extraction conditions, enrichment factors from 117 to 220 were obtained. Recoveries of CPs in various matrices exceed 85% with relative standard deviations of less than 10%, except for PCP. The limits of detection ranged from 0.08 to 2 µg·L^−1^. Table 1 summarises some processes for the determination of phenolic compounds in aqueous samples using conventional extraction methods.

**Table1:** Methods for the determination of phenolic compounds in liquid samples using organic solvents.

Matrix	Extraction technique	Characteristics	Recoveries (%)	LOD (µg·L^-1^)	Instrumental Analysis	Ref.
Tap, river water	On-line SPE	PPy; acetonitrile	84–96	0.03–0.150	LC-UV-Vis	[25]
River water	SPE	Oasis MAX, *n*-hexane	81–116	0.002–0.016	GC-MS	[23]
River water	SPE	OASIS; acetonitrile, dichlorometane	60–98	0.009–0.03	GC-MS/SIM	[35]
River water	SPE	SDS-alumina (admicelles), acetonitrile	60–91	50–1000	LC-UV-Vis	[27]
River, industrial waste water	On-line SPE	Hysphere-GP, acetonitrile, methanol	67–129	0.05–0.10	LC-DAD-EC	[24]
Surface, reused water	SPE	LiChrolut EN, acetone	74–92	20–82	CE-CL	[33]
Well, tap, river water	SPE	Bond Elute PPL, acetone	25–83	0.0005–0.1	GC-ECD	[32]
River, waste water	SPME	PA, methanol	-	2–4 0.017–0.05	LC-UV-Vis and LC-ED	[45]
Waste water	HS-SPME	PDMS-CAR-DVB	-	16–22	GC-MS/SIM	[49]
Sewage water	HS-SPME	PDMS, CAR-PDMS	-	0.001–0.054	HS-GC-MS	[44]
Landfill leachates	SPME	PA,	65–98	0.005–2.5	GC-MS	[53]
Ground water	LPME	Accurel Q3/2 Polypropylene	91–110	0.08–2.01	GC-MS	[59]

PPy: polypyrrole; SDS: Sodium dodecylsulfate

### Solid sample preparation

Although most attention has been focused on the determination of phenolic compounds in aqueous samples, more substituted phenols, such as pentachlorophenol, show limited transport in water and they are more likely absorbed in sediments and soils. This fact contributes to the persistent of these compounds in the environment and it results in high concentrations of them that could affect aquatic and earth organism.

For extraction, Soxhlet extraction is one of the most popular techniques for isolating phenolic compounds from solid samples. This is probably due to its simplicity, inexpensive extraction apparatus and its use in the US EPA official methods, such as 3540 B [21]. Polar solvents, such as methanol, acetone or acetonitrile, give high extraction efficiencies. However, they also extract other undesirable polar compounds present in the samples. With apolar solvents, such as n-hexane or dichloromethane, the extraction of phenols requires a previous acidic digestion of the analytes. Satisfactory recoveries of phenols from soils and sediments have been reported with this technique using, in most cases, mixtures of polar and apolar organic solvents [46,60,61,62,63]. Alonso *et al*. developed an analytical protocol for the determination of priority phenolic compounds in soil samples using a solvent mixture, methanol–water (4:1), both containing 2% triethylamine, to enhance the extraction of more chlorinated phenols [64]. Recoveries varied in the range from 67 to 97% with a standard deviation between 8 and 14%. Despite the results obtained with this methodology, Soxhlet extraction makes the analysis procedure excessive time consuming. Moreover, it requires large amount of hazardous organic solvents.

Ultrasonic extraction is another conventional technique to extract analytes from solid samples. Although sonication is faster than Soxhlet extraction, it also requires large volumes of toxic and expensive organic solvents. Li *et al*. applied this technique followed by SPME and GC-FID to extract chlorophenols from soil samples [65]. Recoveries ranged from 81 to 99 % and RSD were lower than 7%.

In this last decade, microwave energy has been investigated and widely applied in analytical chemistry to accelerate sample digestion, to extract analytes from matrices and in chemical reactions. Microwave energy is a non-ionizing radiation that causes molecular motion by migration of ions and rotation of dipoles, without changing the molecular structures if the temperature is not too high. Nonpolar solvents, such as hexane and toluene, are not affected by microwave energy and, therefore, it is necessary to add polar additives. Microwave-assisted extraction (MAE) is an efficient extraction technique for solid samples which is applicable to thermally stable compounds. Since its development, MAE has became a viable alternative to conventional methodologies due to it has many substantial improvements over other sample preparation techniques such as shorter extraction time, lower amount of solvent and multiple samples analysed at the same time.

In the last few years, the number of papers using this extraction method has increased considerably [66,67,68,69,70]. Many of these publications have evaluated the variables for the optimisation of the MAE procedure and most of them made use of the experimental design approach for the parameters optimisation. The most commonly studied are temperature, extraction time and power, solvent volume and concentration of different solvent mixtures. In the case of phenolic compounds, solvent mixtures such as acetone-hexane and acetone-methanol are usually employed.

Among the parameters that have showed a greatest influence on the extraction efficiency are the microwave temperature and the percentage of acetone in the solvent mixture. This fact is probably due to the differential microwave energy absorption by the solid sample and/or the solvent. When the total solvent volume is high compared with the solid sample volume and contains high proportion of a microwave non-transparent cosolvent, such as acetone, energy should be mostly absorbed by the supernatant solvent rather by the solid sample material. Therefore, heating of the sample is produced not only by direct interaction with microwaves but mainly by convective heating from the top hot solvent mixture layer, thus showing slower extraction kinetics. On the other hand, for solvent mixtures containing low proportions of acetone, convective heating is negligible and microwave energy has to be absorbed by the solid material because the extraction solvent appears to be mostly transparent to microwaves. When low volumes of solvent are used, most of the liquid is in direct contact with the solid material.

A combination of two different techniques, microwave-assisted extraction (MAE) coupled to headspace solid-phase microextraction (SPME-HS), was applied by Wei et al. to determine chlorophenols in soil samples [71]. After analytes were desorbed from SPME fiber in the GC injection port, they were analysed by GC-EC detection system. Under medium power irradiation for 9 minutes, with a polyacrylate fiber, phenolic compounds were extracted with an efficiency about 90% and less than 10% RSD. A different approach, based on microwave-assisted steam distillation (MASD), was used by Ganeshjeevan *et al*. for extracting chlorophenols from solid samples [72]. Gas chromatography (GC) with electron-capture detection was used for the analysis. MASD achieved recoveries for spiked soil samples in the range of 94-101%. Some real samples were analysed which included soil, wood, leather, textiles, dyes and certified reference materials of soil and wood samples. Limit of detection values of 12 ng·g^-1^ for pentachlorophenol and 194 ng·g^-1^ for monochlorophenol were found.

Table 2 shows a summary of procedures used for the determination of phenolic compounds in solid samples using conventional extraction methods.

**Table 2:** Methods for the determination of phenolic compounds in solid samples using organic solvents.

Matrix	Extraction technique	Characteristics	Recoveries (%)	LOD (µg·g^-1^)	Instrumental Analysis	Ref.
Soil	Soxhlet	Acetone, *n*-hexane	-	-	GC-MS (SIM)	[46]
Soil	Soxhlet	Methanol	83–97	-	LC-UV Vis	[64]
Soil	MAE	Methanol	53–92	0.03–0.08 0.02–0.55	LC-UV andLC-APCI-MS(SIM)	[64]
Soil	MAE	Acetone, *n*-hexane	32–78	0.010–0.025	GC-MS	[61]
	Ultrasound agitation		81–99	0.005–0.276	GC-FID	[65]
Soil	MAE-HS-SPME	PA, H_2_O	86–98	0.0001–0.002	GC-ECD	[71]
Soil	MAE-SPE	C_18_, acetic anhydride, triethylamine	94–97	0.01–0.2	GC-ECD	[72]
Sludge, sediments	MAE	Methanol, acetone	78–106	0.0001–0.0003	GC-MS/MS	[68]
Wood, leather, textiles	MAE-SPE	C-18, acetic anhydride, triethylamine	100–106	0.01–0.2	GC-ECD	[72]

## New trends in extraction methods without using organic solvents

The establishment of new analytical methods which improve quality and sensitivity in the determination of environmental pollutants, in liquid and solid samples, is one of the main lines of research in environmental chemistry. These new methodologies must be able to achieve the low concentrations which have been established by the new environmental directives for priority pollutants. Moreover, they must be fast enough and respectful to the environment.

Among these new methods, extraction and preconcentration of organic compounds, such as phenolic compounds, using aqueous solutions of surfactants are a real alternative to conventional methods. The use of surfactants solutions offer several advantages over organic solvent like extractant, such as reduction of solvent amount usage, low cost, easy handling and non-toxic procedures.

It is well known that surfactants, or surface active agents, have the capability to solubilise different kind of solutes as they are constituted by amphiphilic molecules. Surfactant molecules can associate in aqueous solutions to form molecular aggregates called micelles. The minimum concentration of surfactant concentration required for this phenomenon to occur is called the critical micellar concentration (CMC) [73].

Among the different types of surfactants, the non-ionic ones have been widely used to extract organic substances from diverse types of samples [74,75,76,77,78,79,80] which demonstrates their high potential as extractants. Moreover, they are compatible with aqueous–organic mobile phase in chromatographic analysis, which facilitates these applications.

### Liquid sample preparation

The capacity of micelles to solubilise different compounds has been used to develop the extraction and the preconcentration of organic compounds. Watanabe and coworkers introduced the cloud-point technique (CPE) as an alternative and promising technique [73] to the conventional liquid-liquid extraction. This methodology is based on the surfactant capacity to establish two different phases in function of temperature, surfactant concentration, equilibration time, salts addition or acid addition: a surfactant rich phase and aqueous phase, which allows to extract and to preconcentrate the analytes in one step prior their determination, obtaining preconcentration factors depending on the volume relation between surfactant rich phase and aqueous phase [78,79]. The extraction efficiency of relatively apolar organic compounds may reach 100% even when very low surfactant concentrations are used. For more polar species the surfactant concentrations to be used is a critical variable since, on one the hand, it determines the extraction yield and, on the other, it governs the volume of the surfactant-rich phase, which affects the magnitude of the preconcentration factor.

The preconcentration of several chlorophenols has been reported [81] using the non-ionic surfactant C_8_E_3_, polyoxyethylene glycol monooctyl ether, with good extraction yields, although no analytical characteristics were given in that work. Additionally, the ionic surfactant mixture Cetrimide has been used to extract chlorophenols from river water [82]. Phase separation is achieved by the ‘salting-out phenomenon’, adding salts under saturated conditions to the surfactant solution. This approach is compatible with UV–visible detection, but the limits of detection for the different chlorophenols are relatively high, ranging from 27 to 62 mg·L^-1^.

A simple and rapid HPLC method with diode array detection to determine phenolic compounds in water, including the eleven EPA priority pollutants, was developed by Mahugo *et al*. [83,84]. This methodology based on the cloud point phenomenon using non-ionic surfactants was applied to the determination of a phenolic compound mixture in sea water and depurated waste water samples. Under optimum conditions the recoveries obtained were greater than 70% for most of compounds studied and detection limits were lower than 10 μg·L^-1^.

Another non-ionic surfactant, Triton X-114, was used by Calvo Seronero *et al*. to evaluate the potential of the cloud point methodology for the preconcentration of relatively polar compounds using five EPA chlorophenols as test analytes [85]. Analyte determination was performed using reversed-phase gradient LC with electrochemical and spectrophotometric detection. In this study, for the most hydrophobic analyte, pentachlorophenol, the extraction yield was close to 100%, even when low surfactant concentrations were used: 0.05% Triton X-114. However, surfactant concentrations higher than 1% were required to obtain complete extraction of most hydrolytic phenols.

**Figure 2:**
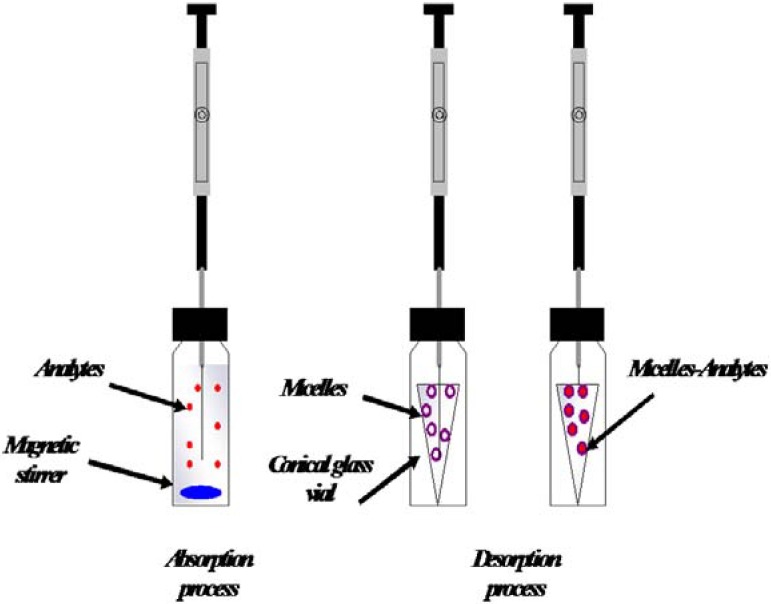
Scheme of solid-phase microextraction with micellar desorption (from Ref. [50]).

Surfactants could be combined with other extraction process like SPME. Normally, desorption process in SPME-HPLC may be made in an appropriate desorption chamber, using an organic solvent or using off-line organic solvent desorption. SPME–HPLC has been used to extract and preconcentrate chlorophenols, mainly from water samples [45,52], but desorption of analytes in the fiber desorption chamber has some disadvantages. When elution is performed in the static mode because of the low volume of the desorption chamber, analytes are not totally desorbed at the first elution, so they partially remained in the fiber and most of them eluted in subsequent desorptions. Dynamic desorption usually results in broad chromatographic peaks due to slow desorption of the analytes from the fiber into the mobile phase. For that, SPME–HPLC has limited application due to the optimization of desorption conditions. Mahugo *et al*. have developed a solid phase microextraction method with a new desorption mode using a micellar medium as desorbing agent (SPME-MD) and combined with HPLC (SPME-MD-HPLC) with diode array detection (DAD) to achieve greatest efficiencies in the extraction [86]. This process has been implemented to the extraction of chlorophenols from environmental liquid samples. A scheme of the process is shown in Figure 2. In this procedure, the desorption is carried out using the non-ionic surfactant polyoxyethylene 10 lauryl ether (POLE) in a 100 µL conical glass vial to avoid the disadvantages that are present in the desorption chamber using organic solvent, improving the desorption process. A satisfactory reproducibility for the extraction of target compounds, between 6 and 15%, was obtained, and detection limits were in the range of 1.1–5.9 µg·L^−1^. Detection limits were enhanced in relation to conventional SPME-HPLC method [45,51,52]. The application of the SPME-MD-HPLC method in different types of spiked water samples showed better efficiency in the analytes desorption with micellar media. The SPME-MD-HPLC method was applied by Torres *et al*. to the extraction of EPA priority phenols from water samples [50]. Data obtained confirmed that the use of non-ionic surfactants enhances the desorption efficiency and it increases with the surfactant polarity. Recoveries for the target compounds extracted from different kinds of spiked water samples were between 80 and 109% and detection limits were in the range 0.3–3.5 µg·L^-1^.

### Solid sample preparation

Normally, analytes extraction from solid samples requires a long contact time between the sample and the extractant. Use of microwave reduces the extraction time. MAE is a technique with several advantages compared with traditional ones, as short analysis times, low extraction volumes and multiples analysis in one run. Use of surfactants in microwave extraction represents an alternative to organic solvents. Microwave assisted micellar extraction (MAME) reduces also method’s toxicity, doing it an environmental friendly extraction method.

Microwave-assisted micellar extraction has been optimised and applied to the extraction, prior to analysis by liquid chromatography with diode array detection, of chlorophenols in marine sediment samples [87]. This study was carried out using a non-ionic surfactant, polyoxyethylene-6-lauryl ether, as extractant. Parameters studied included surfactant concentration, pH of the solution, extraction time and power. The optimised method was applied to different spiked marine sediments. Detection limits were obtained in the range 1.2–12.7 µg·g^−1^ for the chlorophenols studied. The proposed method provides a simple, fast and organic solvent-free procedure to analyse for chlorophenols in marine sediment samples.

**Figure 3:**
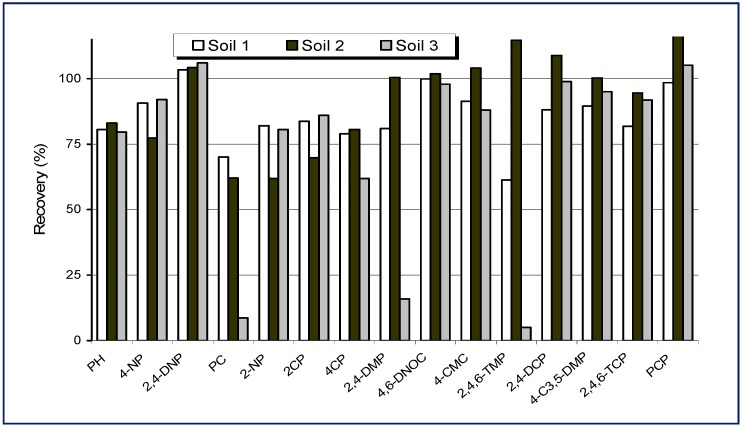
Recoveries obtained for phenolic compounds in three different soil samples after MAME procedure (from Ref. [88]).

A different non-ionic surfactant, polyoxyethylene 10 lauryl ether (POLE), has been used as extractant for the microwave-assisted micellar extraction (MAME) of phenolic compounds from soil samples of different characteristics in order to investigate the matrix effect in the extraction efficiency [88]. Fifteen phenolic compounds, including the priority, and three different types of soil were studied. The influence of soil pH on phenol extraction was studied using acid soils from a pine forest (soil 1) and a grow soil (soil 2), and an alkaline one from a garden (soil 3). Figure 3 shows the results obtained. It can see that the recoveries of 13 phenols were higher than 80% in the acid soils, whereas the values obtained in the alkaline soil were slightly lower. Furthermore, the alkylphenols were extracted satisfactorily, but their recoveries are lower than those obtained in the samples that are high in organic matter (soil 2).

The texture of a soil is extremely important in the sorption process. Clay is by far the most adsorbent of the three main soil textures (clay, silt, and sand) due to its small particle size, high surface area, and high surface charge. Although the soils tested in this study were sandy ones, soil 1 had a higher amount of particles with a smaller size. The results (Figure 3) indicate that for these soil types, with high sand content, the soil texture is the parameter that has greater influence on the sorption process than the soil pH or organic matter content. On the other hand, taking into account that the surfactants have a high capacity to extract humic and fulvic acids and that the phenols can be adsorbed into organic matter, this fact may explain the high recoveries of the compounds extracted from the soil with a higher organic matter content (soil 2).

In order to study the aging effects, MAME procedure was applied to the three soils for different time periods after conditioning. The analytes present in recent soils samples are more easily extracted than those that have had a longer contact time. This can be explained according to whether the analytes are incorporated by adsorption (short periods) or by sequestration (longer periods) [89].

**Figure 4:**
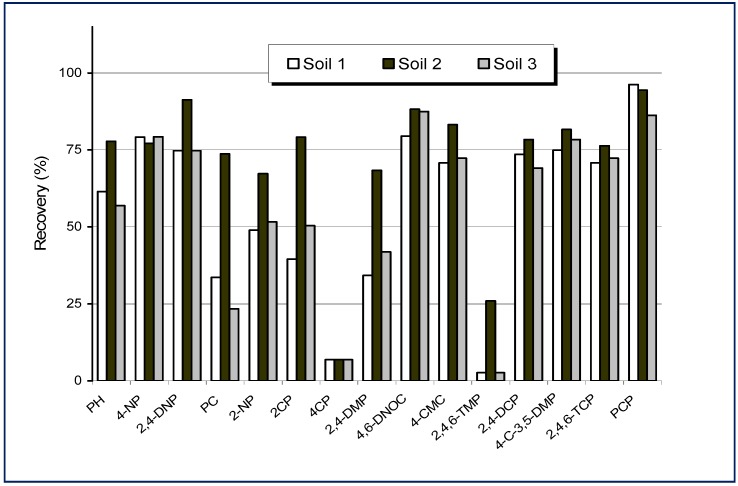
Recoveries obtained for phenolic compounds in three different soil samples for eight weeks after conditioning after the MAME procedure (from Ref. [88]).

Results obtained with the proposed method achieved good recoveries for aged samples, except for alkylphenols. Figure 4 illustrates the recoveries obtained from samples spiked eight weeks before analysis. A new approach, using microwave-assisted micellar extraction in combination with the solid-phase microextraction (MSPME), has been used to determine chlorophenols in wood samples [90]. Variables optimisation of the focused-microwave system and the effect of the aged time of the samples in the extraction efficiency of the method have been assessed in this study. The overall method using the non-ionic surfactant POLE as extracting gave an average extraction efficiency of 104%, limits of detection ranging from 2 to 120 ng·g^−1^ and intermediate precision values ranging between 3.5 and 13.2%. This technique has been successfully applied to the determination of analyte–micelle partition coefficients for PAHs and phenols [91,92] or to study interactions between phenols and different micellar media [93]. Table 3 summarises some processes for the determination of phenolic compounds in aqueous and solid samples using micellar media as extractant agents.

**Table 3:** Methods for the determination of phenolic compounds using surfactant solutions.

Matrix	Extraction technique	Surfactants	Recoveries (%)	LOD	Instrumental Analysis	Ref.
Sea water waste water	CPE	Oligoethylene glycol monoalkyl ether (Genapol X-080)	66-119	1.0-5.0 (µg.L^-1^)	LC-UV	[83]
Sea water waste water	CPE	Polyoxyethylene 10 lauryl ether (POLE) Polyoxyethylene 9 lauryl ether (Polidocanol) Polyoxyethylene 6 lauryl ether (C_12_E_6_)	44-115	0.6–3.5 (µg.L^-1^)	LC-UV	[84]
Water	CPE	Poly(oxyethylene)-7,5-( *p*-*tert*-octylphenyl) ether (Triton X-114)	62-101	2.0-5.0 (µg.L^-1^)	LC-UV-EC	[85]
River water lake water	CPE	Triton X-114	93-103	2.0-2.5 (mol.L^−1^)	CE-UV	[95]
Sediments	MAME	Polyoxyethylene-6-lauryl ether	81-120	1.2–12.7(µg.g^−1^)	LC-UV	[87]
Soil	MAME	POLE	70-118	–	LC-UV	[88]
Sediments	MAME	(Polidocanol) Oligoethylene glycol monoalkyl ether (Genapol X-080)	79-117	2-20 (µg.g-1)	LC-UV	[94]
Sea water, sewage water ground water	SPME-MD CW-TPR	POLE	89-107	1.1-5.9 (µg.L^-1^)	LC-UV	[86]
Sea water, sewage water ground water	SPME-MD CW-TPR PDMS PDMS-DVB PA Carboxen-PDMS	POLE, Polidocanol, Polyoxyethylene 6 lauryl ether (C_12_E_6_) Hexadecyltrimethylammonium bromide (HTAB)	80-109	0.3–3.5(µg.L^-1^)	LC-UV	[50]
Wood	MAME-MSPME PA	POLE	71-125	0.002-0.12 (µg.g-1)	GC-MS	[90]

## Conclusions

Sample preparation is one of the most critical steps in the determination of phenolic compounds in different environmental matrices. In the last years, there has been a notable increase in the amount of the literature related with novel sample treatment techniques applied to these compounds in environmental samples. Surfactant solutions have been used like an alternative to the use of organic solvents. Although techniques such as SPME in liquid samples and MAE in solid samples have been extensively used, micellar solutions have demonstrated to play an important role in these new treatments. Combination of surfactant solutions with them improves considerably the quality of analytical methods used. Their main characteristics are less use of organic solvents, smaller volume samples and less sample preparation, between them. Moreover, surfactants are less toxic, easy to handle and cheaper than other solvents. Analysis of compounds can be performed by chromatographic techniques. Future research in this area should be focused toward improvements of the extraction and determination of phenolic compounds combining SPE and SPME with micellar solutions with more sensitive techniques (e.g. LC-MS).
